# Characterizing the HIV risks and potential pathways to HIV infection among transgender women in Côte d'Ivoire, Togo and Burkina Faso

**DOI:** 10.7448/IAS.19.3.20774

**Published:** 2016-07-17

**Authors:** Shauna Stahlman, Benjamin Liestman, Sosthenes Ketende, Seni Kouanda, Odette Ky-Zerbo, Marcel Lougue, Daouda Diouf, Simplice Anato, Jules Tchalla, Amara Bamba, Fatou Maria Drame, Rebecca Ezouatchi, Abo Kouamé, Stefan D Baral

**Affiliations:** 1 Department of Epidemiology, Johns Hopkins Bloomberg School of Public Health Baltimore, MD, USA; 2 Institut de Recherche en Sciences de la Santé, Ouagadougou, Burkina Faso; 3 Institut Africain de Santé Publique, Ouagadougou, Burkina Faso; 4 Programme d'Appui au Monde Associatif et Communautaire, Ouagadougou, Burkina Faso; 5 Enda Santé Senegal, Dakar, Sénégal;; 6 Arc-en-ciel, Lomé, Togo; 7 Espoir Vie, Lomé, Togo; 8 Enda Santé, Abidjan, Côte d'Ivoire; 9 Programme National de Lutte contre le Sida, Ministère de la Santé et de la Lutte contre le Sida, Abidjan, Côte d'Ivoire

**Keywords:** HIV, transgender women, stigma, sub-Saharan Africa, epidemiology, sexual risk behaviours, structural equation modelling

## Abstract

**Introduction:**

Transgender women are at high risk for the acquisition and transmission of HIV. However, there are limited empiric data characterizing HIV-related risks among transgender women in sub-Saharan Africa. The objective of these analyses is to determine what factors, including sexual behaviour stigma, condom use and engagement in sex work, contribute to risk for HIV infection among transgender women across three West African nations.

**Methods:**

Data were collected via respondent-driven sampling from men who have sex with men (MSM) and transgender women during three- to five-month intervals from December 2012 to October 2015 across a total of six study sites in Togo, Burkina Faso and Côte d'Ivoire. During the study visit, participants completed a questionnaire and were tested for HIV. Chi-square tests were used to compare the prevalence of variables of interest between transgender women and MSM. A multilevel generalized structural equation model (GSEM) was used to account for clustering of observations within study sites in the multivariable analysis, as well as to estimate mediated associations between sexual behaviour stigma and HIV infection among transgender women.

**Results:**

In total, 2456 participants meeting eligibility criteria were recruited, of which 453 individuals identified as being female/transgender. Transgender women were more likely than MSM to report selling sex to a male partner within the past 12 months (*p<*0.01), to be living with HIV (*p<*0.01) and to report greater levels of sexual behaviour stigma as compared with MSM (*p<*0.05). In the GSEM, sexual behaviour stigma from broader social groups was positively associated with condomless anal sex (adjusted odds ratio (AOR)=1.33, 95% confidence interval (CI)=1.09, 1.62) and with selling sex (AOR=1.23, 95% CI=1.02, 1.50). Stigma from family/friends was also associated with selling sex (AOR=1.42, 95% CI=1.13, 1.79), although no significant associations were identified with prevalent HIV infection.

**Conclusions:**

These data suggest that transgender women have distinct behaviours from those of MSM and that stigma perpetuated against transgender women is impacting HIV-related behaviours. Furthermore, given these differences, interventions developed for MSM will likely be less effective among transgender women. This situation necessitates dedicated responses for this population, which has been underserved in the context of both HIV surveillance and existing responses.

## Introduction

Transgender women, defined here as individuals who were assigned the male sex at birth but who identify as women, are at high risk for HIV acquisition and transmission [1,2]. Globally, the odds of being infected with HIV for transgender women are almost 50 times those of other adults of reproductive age, with a pooled HIV prevalence of around 19% [1]. Even compared to other key populations such as men who have sex with men (MSM), transgender women appear to be at increased risk for HIV transmission [1,3]. A primary driver of this burden, similar to MSM, is the high transmission probability of condomless anal sex [4]. Transgender women also experience high levels of multiple intersecting stigmas, such as stigma related to gender identity, sexual practices, sex work and HIV status [5–10]. Sexual behaviour stigma, which is defined here as stigma that is anticipated, perceived or experienced as a result of one's sexual experience, is just one form of stigma that may be shared in common between MSM and transgender women [11]. However, because of the potential non-additive effects of these intersecting stigmas, the negative health outcomes due to sexual behaviour stigma may be even more severe for transgender women than for MSM [12,13].

A qualitative study of transgender women in the United States provided one example of the impact of stigma and discrimination on increasing the risk for HIV infection. The study noted that stigma can reduce self-esteem within the context of romantic relationships, which can lead to reduced condom use for the sake of these women pleasing their partner [14]. Sexual behaviour stigma and gender-related abuse have also been associated with risk factors for HIV infection, including condomless anal sex [8,15,16], as well as reduced access to/uptake of HIV prevention and care services [3,17,18]. Because transgender women are often among the most marginalized and economically discriminated against in societies, many engage in sex work to support themselves [19,20], which can further increase the risk for HIV transmission [19].

In Togo, Burkina Faso and Côte d'Ivoire, the HIV prevalence among adults aged 15 to 49 in 2014 ranged from 1 to 3.5% [21]. However, MSM communities in these countries experience a much greater burden of infection, with HIV prevalences ranging from 10 to 35% [22]. In addition, there are almost no empiric data on the risk factors for HIV infection among transgender women anywhere on the African continent, including West Africa [1,3,23]. Historically and also in the context of the HIV/AIDS epidemic, most have denied or downplayed the existence of gay, lesbian, bisexual and transgender individuals in Africa [24]. The lack of research on transgender populations in countries across sub-Saharan Africa has contributed to lack of funding and clinical competency for transgender-specific HIV services [23]. Although visibility is increasing, the increased exposure to violence and victimization among transgender women has also contributed to the persistent lack of epidemiological research in this group [23]. As a result, we know little about sexual practices, HIV prevalence and risk factors among transgender women in the region.

The objective of these analyses is to determine what factors, such as sexual behaviour stigma, condom use and engagement in sex work, contribute to the risk for HIV infection among transgender women across three West African nations (Burkina Faso, Côte d'Ivoire and Togo). By studying the potential effects of sexual behaviour stigma across gender identities, we utilize intersectional research methods with the goal of unifying theoretical frameworks of stigma to reduce health disparities among transgender women, and to support data that examine multiple social identities (gay men and other MSM, as well as transgender women who have sex with men) [12,25,26]. During the period of data collection, same sex practices were illegal in Togo [27]. Although not criminalized in Côte d'Ivoire and Burkina Faso, same sex practices remained highly stigmatized and without any constitutional protections against discrimination [27,28]. Specifically, we explored the HIV risk factors that affect transgender women as compared with MSM and assessed which factors could potentially mediate the association between sexual behaviour stigma and risk of HIV infection.

## Methods

### Study population and sampling methods

Data were collected as part of larger cross-sectional studies including quantitative questionnaires, HIV testing and population size estimates of MSM in several West African countries. The studies took place from December 2012 to June 2013 (Kara and Lome) in Togo; January to August 2013 (Bobo-Dioulasso and Ouagadougou) in Burkina Faso; and in Côte d'Ivoire in March to May 2015 (Abidjan), July to September 2015 (Bouaké), May to June 2015 (Gagnoa) and September to October 2015 (Yamoussoukro). The study cities were chosen based on the following criteria: large enough to enable recruitment of the proposed sample size (all urban cities), far enough away from other cities so that the same population was not resampled, representative of different areas and cultures within the country, and existing relationships of trust with activities and programmes that work with MSM. Eligible participants had to be at least 18 years old, to have been assigned male sex at birth, to be capable of providing informed oral consent, and to report having had insertive or receptive anal sex with a man in the past 12 months. During the questionnaire, participants were asked, “What do you consider your gender to be?” Those responding “female” or “transgender” were considered as transgender women for these analyses. The response options were “man,” “woman” or “intersex” in Togo and Burkina Faso and “man,” “woman,” “transgender” or “other (specify)” in Côte d'Ivoire. *Intersex* was explained to participants as meaning that they did not identify as either male or female, and key informants indicated that participants interpreted “transgender” as meaning the same thing as “female.”

Participants were recruited using respondent-driven sampling (RDS) [29,30]. To begin recruitment, three to six *seeds*, or initial recruits, were selected based on the recommendation of local community-based organizations, representing a range of characteristics including age, education, socio-economic status and participation in LGBT associations. In Bobo-Dioulasso, 24 waves of accrual were reached, with all other sites reaching between 7 and 14 waves. Equilibrium was reached for the outcome variable of interest (HIV status) and for sociodemographics (e.g., age, education) in all study sites. Equilibrium was defined as the point at which the cumulative sample proportions came within 2% of the final sample proportion and did not fluctuate more than 2% during the sampling of additional waves [31].

Participants were reimbursed for the cost of travel to the study site. In Togo, participants were additionally reimbursed for the cost of one meal. RDS recruiters were compensated the equivalent of US$3 (Burkina), US$6 (Togo) and US$2 (Côte d'Ivoire) for each eligible participant they recruited into the study (up to three recruits). Studies were approved by the Ethical Committee of Togo, the Health Research Ethics Committee of Burkina Faso, the Health Research Ethics Committee of Côte d'Ivoire and the institutional review board at the Johns Hopkins Bloomberg School of Public Health.

## Data collection and key measures

During the study visit, trained interviewers administered a structured questionnaire including modules on demographics, sexual risk practices and sexual behaviour stigma. Interviews were conducted in French or in the local languages of Ewe or Kabiyè (Togo) or Mooré or Dioula (Burkina Faso), by interviewers who were fluent and trained in conducting the interviews in the local language of the city.

Sexual risk practices included the number of receptive anal sex partners within the past 12 months (Togo and Burkina) and number of regular receptive anal sex partners within the past 30 days (Côte d'Ivoire; number of casual receptive anal sex partners was not measured). Participants were also asked whether they used a condom during the last time they had anal sex with a male partner. Sexual positioning variables were generated for those who only engaged in receptive anal sex (“bottoms”), those who only engaged in insertive anal sex (“tops”) and those who engaged in both receptive and insertive anal sex (“versatiles”). In order to measure engagement in sex work, participants were asked whether they had anal or oral sex with any men in the last 12 months in exchange for things they wanted or needed such as money, drugs, food, shelter or transportation.

Sexual behaviour stigma measures consisted of a series of *yes*/*no* questions that assessed four domains [32–35]. Domains were identified via exploratory factor analysis using tetrachoric correlations. They included the following: 1) stigma from family and friends (e.g., “Have you ever felt excluded from family gatherings because you have sex with men?”), 2) perceived healthcare stigma (e.g., “Have you ever felt afraid to go to healthcare services because you worry someone may learn you have sex with men?”), 3) experienced healthcare stigma (e.g., “Have you ever heard healthcare providers gossiping about you because you have sex with men?”) and 4) social stigma (e.g., “Have you ever been verbally harassed and felt that it was because you have sex with men?”). Social stigma also included whether the participant had ever been physically attacked or forced to have sex and felt that it was because they have sex with men. Of the 13 initial items, two were removed due to cross loading on two or more factors. Four stigma domain variables were created for use in the multivariable model, which consisted of the sum of the total number of *yes* responses for each domain.

At the end of the survey, participants were tested for HIV using the Alere Determine HIV 1/2 Ag/Ab Combo Rapid Test (Waltham, MA, USA). If there was a positive result, either the HIV Bispot ImmunoComb II (Orgenics, Yavne, Israel), First Response HIV 1-2.O Card Test (Premier Medical Corporation, Nani Daman, India) or Clearview HIV 1/2 STAT-PAK™ (Chembio Diagnostic Systems, Medford, NY, USA) were used to confirm the result in Burkina Faso, Togo, or Cote d'Ivoire, respectively. The specificity for the HIV confirmatory test was 100% in all settings. Participants who tested positive for HIV at any of the study sites were provided referrals for treatment.

### Statistical analysis

Because we combined data from multiple study sites to maximize the number of transgender women, no adjustments were made for RDS sampling methods [36]. Chi-square tests were used to compare the prevalence of variables of interest between transgender women and MSM both overall and stratified by country. A multilevel generalized structural equation model (GSEM) (Stata 14, StataCorp, College Station, TX, USA) was used to account for clustering of observations within study sites by including a latent variable at the study site level in the multivariable analysis, as well as to estimate mediated associations between sexual behaviour stigma and HIV infection among transgender women in the combined data set. The model adjusted for age and education level as potential confounders based on previous knowledge [16,37,38] and on results from bivariate analyses. Participants with missing data for variables of interest were excluded from the analyses.

## Results

### Study sample

In all, 2456 eligible participants were recruited across the six study sites, including 453 (18.4%) individuals who identified as being female or transgender. Those who reported being intersex (*n=*208) or both male and female (*n=*1) or “don't know” (*n=*1) were not considered to be transgender women nor MSM and were excluded from these analyses *post hoc*. The median age of the participants was 23 years, most had completed secondary/high school education (65%) and a large proportion were students (49%). Transgender women were similar to MSM in terms of employment status (*p=*0.07) (Table 1). However, transgender women were more likely to only have completed primary school or a lower level of education (*p<*0.01) and were slightly younger (*p<*0.05). In addition, transgender women were disproportionately sampled across certain study sites, with the largest percentages of transgender women attending study locations in Abidjan (31%) and Bobo-Dioulasso (17%). These sociodemographic trends were similar when we examined associations stratified by country, although transgender women were less likely than MSM to be students as compared with employed or unemployed in Burkina Faso (*p<*0.01) (Tables 2–4).

**Table 1:** Prevalence of sociodemographic characteristics of transgender women as compared with MSM participants in three West African nations (*N=*2246)

	Total		MSM		Transgender women		
				
Characteristic	*n*	%	*n*	%	*n*	%	χ^2^*p*^a^
Median age (IQR)^b^	23 (21 to 26)		23 (21 to 26)		22.5 (20 to 26)		0.02*
Education completed							
Primary school or lower	211	9.5	139	7.8	72	16.1	<0.001***
Secondary/high school	1437	64.5	1174	66.0	263	58.7	
More than high school	579	26.0	466	26.2	113	25.2	
Employment status							
Unemployed	238	10.6	187	10.4	51	11.3	0.07
Student	1102	49.1	902	50.3	200	44.3	
Employed	905	40.3	704	39.3	201	44.5	
Study site location							
Bobo-Dioulasso, Burkina Faso	276	12.3	201	11.2	75	16.6	<0.001***
Ouagadougou, Burkina Faso	265	11.8	242	13.5	23	5.1	
Abidjan, Côte d'Ivoire	350	15.6	211	11.8	139	30.7	
Bouake, Côte d'Ivoire	350	15.6	287	16.0	63	13.9	
Gagnoa, Côte d'Ivoire	150	6.7	113	6.3	37	8.2	
Yamoussoukro, Côte d'Ivoire	250	11.1	183	10.2	67	14.8	
Kara, Togo	307	13.7	301	16.8	6	1.3	
Lome, Togo	298	13.3	255	14.2	43	9.5	

* *p<*0.05

** *p<*0.01

*** *p<*0.001

a *p*-value derived using Pearson's chi-square test

b *p*-value derived using the Wilcoxon rank-sum test; MSM, men who have sex with men.

**Table 2:** Prevalence of sociodemographic characteristics of transgender women as compared with MSM participants in Togo (*N=*605)

	Total		MSM		Transgender women		
				
Characteristic	*n*	%	*n*	%	*n*	%	*χ*^2^*p*^a^
Median age (IQR)^b^	23 (21 to 26)		23 (21 to 26)		21 (20 to 25)		0.004**
Education completed							
Primary school or lower	32	5.3	26	4.7	6	12.2	0.04*
Secondary/high school	410	68.0	376	67.9	34	69.4	
More than high school	161	26.7	152	27.4	9	18.4	
Employment status							
Unemployed	62	10.3	59	10.6	3	6.1	0.08
Student	215	35.5	203	36.5	12	24.5	
Employed	328	54.2	294	52.9	34	69.4	
Study site location							
Kara	307	50.7	301	54.1	6	12.2	<0.001***
Lome	298	49.3	255	45.9	43	87.8	

* *p*<0.05

** *p*<0.01

*** *p*<0.001

a *p*-value derived using Pearson's chi-square test

b *p*-value derived using the Wilcoxon rank-sum test; MSM, men who have sex with men.

**Table 3:** Prevalence of sociodemographic characteristics of transgender women as compared with MSM participants in Burkina Faso (*N=*541)

	Total		MSM		Transgender women		
				
Characteristic	*n*	%	*n*	%	*n*	%	*χ*^2^*p*^a^
Median age (IQR)^b^	22 (20 to 24)		22 (20 to 24)		21 (20 to 24)		0.70
Education completed							
Primary school or lower	51	9.6	38	8.7	13	13.8	0.13
Secondary/high school	384	72.2	315	71.9	69	73.4	
More than high school	97	18.2	85	19.4	12	12.8	
Employment status							
Unemployed	27	5.0	18	4.1	9	9.2	0.002**
Student	344	63.6	296	66.8	48	49.0	
Employed	170	31.4	129	29.1	41	41.8	
Study site location							
Bobo-Dioulasso	276	51.0	201	45.4	75	76.5	<0.001***
Ouagadougou	265	49.0	242	54.6	23	23.5	

* *p<*0.05

** *p<*0.01

*** *p<*0.001

a *p*-value derived using Pearson's chi-square test

b *p*-value derived using the Wilcoxon rank-sum test; MSM, men who have sex with men.

**Table 4:** Prevalence of sociodemographic characteristics of transgender women as compared with MSM participants in Côte d'Ivoire (*N=*1100)

	Total		MSM		Transgender women		
				
Characteristic	*n*	%	*n*	%	*n*	%	*χ*^2^*p*^a^
Median age (IQR)^b^	24 (21 to 27)		24 (22 to 27)		23 (21 to 27)		0.02*
Education completed							
Primary school or lower	128	11.7	75	9.5	53	17.4	<0.001***
Secondary/high school	643	58.9	483	61.4	160	52.5	
More than high school	321	29.5	229	29.1	92	30.2	
Employment status							
Unemployed	149	13.6	110	13.9	39	12.8	0.19
Student	543	49.4	403	50.8	140	45.9	
Employed	407	37.0	281	35.4	126	41.3	
Study site location							
Abidjan	350	31.8	211	26.6	139	45.4	<0.001***
Bouake	350	31.8	287	36.2	63	20.6	
Gagnoa	150	13.6	113	14.2	37	12.1	
Yamoussoukro	250	22.7	183	23.1	67	21.9	

* *p<*0.05

** *p<*0.01

*** *p<*0.001

a *p*-value derived using Pearson's chi-square test

b *p*-value derived using the Wilcoxon rank-sum test; MSM, men who have sex with men.

### Prevalence of HIV risk-related characteristics

Transgender women were more likely than MSM to report recently engaging in exclusively receptive anal sex (*p<*0.01) and less likely to report recently engaging in exclusively insertive anal sex (*p<*0.01) (Table 5). However, overall transgender women were not found to be more or less likely to report sexual position versatility (i.e., practicing both receptive and insertive anal sex). Transgender women were, however, more likely to report condomless anal sex (*p<*0.01), selling sex to a male partner (*p<*0.01) and were more likely to be living with HIV (*p<*0.01) as compared with MSM.

**Table 5:** Prevalence of HIV risk-related characteristics of transgender women as compared with MSM participants in three West African nations (*N=*2246)

	Total		MSM		Transgender women		
				
HIV risk-related characteristic	*n*	%	*n*	%	*n*	%	*χ*^2^*p*^a^
Sexual position^b^							
Insertive only	801	47.9	770	59.4	31	8.2	<0.001***
Receptive only	290	17.3	84	6.5	206	54.6	<0.001***
Versatile	583	34.8	443	34.2	140	37.1	0.29
Condomless anal sex, last anal sex episode	583	26.6	438	24.9	145	33.3	<0.001***
Sold sex to male partner, past 12 months	874	39.2	658	37.0	216	48.1	<0.001***
Living with HIV	201	9.2	119	6.8	82	18.9	<0.001***

* *p<*0.05

** *p<*0.01

*** *p<*0.001

a *p*-value derived using Pearson's chi-square test

b within the past 12 months in Togo/Burkina Faso and within the past 30 days in Côte d'Ivoire; MSM, men who have sex with men.

There was some variation when results were stratified by country (Tables 6–8). Namely, transgender women were less likely than MSM to report sexual position versatility in Togo (*p<*0.05) and were more likely to report sexual position versatility in Côte d'Ivoire (*p<*0.01). Transgender women were not more likely to be living with HIV in Burkina Faso (*p=*0.96), though the overall HIV prevalence was lower there. Transgender women were more likely to report a higher number of receptive anal sex partners in all settings and were more likely to report condomless anal sex in all countries except for Burkina Faso.

**Table 6:** Prevalence of HIV risk-related characteristics of transgender women as compared with MSM participants in Togo (*N=*605)

	Total		MSM		Transgender women		*χ*^2^*p*^a^
			
HIV risk-related characteristic	*n*	%	*n*	%	*n*	%	n
Median number of receptive anal sex partners, past 12 months (IQR)^b^	0 (0 to 1)		0 (0 to 0)		1 (0 to 2)		<0.001***
Sexual position, past 12 months							
Insertive only	101	38.7	95	41.7	6	18.2	0.01*
Receptive only	36	13.8	18	7.9	18	54.6	<0.001***
Versatile	124	47.5	115	50.4	9	27.3	0.01*
Condomless anal sex, last anal sex episode	146	24.3	127	23.0	19	39.6	0.01*
Sold sex to male partner, past 12 months	172	28.5	152	27.4	20	40.8	0.046*
Living with HIV	51	8.5	42	7.6	9	18.8	0.008**

* *p<*0.05

** *p<*0.01

*** *p<*0.001

a *p*-value derived using Pearson's chi-square test

b *p*-value derived using the Wilcoxon rank-sum test; MSM, men who have sex with men.

**Table 7:** Prevalence of HIV risk-related characteristics of transgender women as compared with MSM participants in Burkina Faso (*N=*541)

	Total		MSM		Transgender women		
				
HIV risk-related characteristic	*n*	%	*n*	%	*n*	%	*χ*^2^*p*^a^
Median number of receptive anal sex partners, past 12 months (IQR)^b^	1 (0 to 2)		2 (0 to 2)		3 (2 to 6)		<0.001***
Sexual position, past 12 months							
Insertive only	210	38.8	205	46.3	5	5.1	<0.001***
Receptive only	78	14.4	25	5.6	53	54.1	<0.001***
Versatile	253	46.8	213	48.1	40	40.8	0.19
Condomless anal sex, last anal sex episode	134	24.8	106	24.0	28	28.6	0.34
Sold sex to male partner, past 12 months	207	39.1	164	37.8	43	44.8	0.20
Living with HIV	27	5.0	22	5.0	5	5.1	0.96

* *p<*0.05

** *p<*0.01

*** *p<*0.001

a *p*-value derived using Pearson's chi-square test

b *p*-value derived using the Wilcoxon rank-sum test; MSM, men who have sex with men.

**Table 8:** Prevalence of HIV risk-related characteristics of transgender women as compared with MSM participants in Côte d'Ivoire (*N=*1100)

	Total		MSM		Transgender women		
				
HIV risk-related characteristic	*n*	%	*n*	%	*n*	%	*χ*^2^*p*^a^
Median number of regular receptive anal sex partners, past 30 days (IQR)^b^	0 (0 to 1)		0 (0 to 0)		1 (0 to 2)		<0.001***
Sexual position, past 30 days							
Insertive only	490	56.2	470	75.1	20	8.1	<0.001***
Receptive only	176	20.2	41	6.6	135	54.9	<0.001***
Versatile	206	23.6	115	18.4	91	37.0	<0.001***
Condomless anal sex, last anal sex episode	303	28.8	205	26.9	98	33.9	0.03*
Sold sex to male partner, past 12 months	495	45.2	342	43.2	153	50.3	0.03*
Living with HIV	123	11.7	55	7.3	68	23.5	<0.001***

* *p*<0.05

** *p*<0.01

*** *p*<0.001

a *p*-value derived using Pearson's chi-square test

b *p*-value derived using the Wilcoxon rank-sum test; MSM, men who have sex with men.

### Prevalence of sexual behaviour stigma

Transgender women in the combined sample were more likely than MSM to report feeling excluded by family members (* p<*0.01), gossiped about by family members (*p<*0.01) and rejected by friends (*p<*0.01) (Table 9). For healthcare-related sexual behaviour stigma, a slightly higher prevalence of transgender women reported avoiding seeking healthcare (*p<*0.05) and being treated poorly at a healthcare centre (*p<*0.05). Finally, a greater proportion of transgender women reported being verbally harassed (*p<*0.01), blackmailed (*p<*0.05), physically hurt (*p<*0.01) and raped (*p<*0.01) because of their sexual behaviours.

**Table 9:** Prevalence of sexual behaviour stigma among transgender women as compared with MSM participants in three West African nations (*N=*2246)

	Total		MSM		Transgender women		
				
	*n*	%	*n*	%	*n*	%	*χ*^2^*p*^a^
**Stigma from family/friends**							
Excluded by family	223	9.9	156	8.7	67	14.8	<0.001***
Gossiped about by family	641	28.6	448	25.0	193	42.7	<0.001***
Rejected by friends	501	22.3	358	20.0	143	31.6	<0.001***
**Perceived healthcare stigma**							
Afraid to seek healthcare	473	21.1	364	20.3	109	24.1	0.08
Avoided seeking healthcare	363	16.2	276	15.4	87	19.2	0.049*
**Experienced healthcare stigma**							
Treated poorly at a healthcare centre	60	2.7	41	2.3	19	4.2	0.02*
Gossiped about by healthcare worker	167	7.4	124	6.9	43	9.5	0.06
**Social stigma**							
Verbally harassed	727	32.4	475	26.5	252	55.6	<0.001***
Blackmailed	420	18.7	320	17.9	100	22.1	0.04*
Physically hurt	253	11.3	139	7.8	114	25.3	<0.001***
Raped	230	10.3	131	7.3	99	21.9	<0.001***

* *p<*0.05

** *p<*0.01

*** *p<*0.001

a *p*-value derived using Pearson's chi-square test; MSM, men who have sex with men.

In the analysis stratified by country, transgender women consistently reported higher levels of verbal harassment (Tables 10–12). Transgender women also reported significantly higher levels of physical assault in Côte d'Ivoire (*p<*0.01) and Burkina Faso (*p<*0.01), as well as higher levels of rape in Côte d'Ivoire (*p<*0.01). Perceived and experienced healthcare stigma was not significantly higher among transgender women in Togo or Burkina Faso, and family/friend stigma was not significantly higher among transgender women in Togo. However, in Togo several cell counts for family- and healthcare-related stigma were below 5.

**Table 10:** Prevalence of sexual behaviour stigma among transgender women as compared with MSM participants in Togo (*N=*605)

	Total		MSM		Transgender women		
				
	*n*	%	*n*	%	*n*	%	*χ*^2^*p*^a^
**Stigma from family/friends**							
Excluded by family	51	8.4	46	8.3	5	10.2	0.64
Gossiped about by family	94	15.5	83	14.9	11	22.5	0.16
Rejected by friends	70	11.6	66	11.9	4	8.2	0.64
**Perceived healthcare stigma**							
Afraid to seek healthcare	53	8.8	47	8.5	6	12.2	0.37
Avoided seeking healthcare	42	6.9	40	7.2	2	4.1	0.56
**Experienced healthcare stigma**							
Treated poorly at a healthcare centre	7	1.2	6	1.1	1	2.0	0.45
Gossiped about by healthcare worker	24	4.0	20	3.6	4	8.2	0.12
**Social stigma**							
Verbally harassed	97	16.0	82	14.8	15	30.6	0.004**
Blackmailed	106	17.5	93	16.7	13	26.5	0.08
Physically hurt	21	3.5	17	3.1	4	8.2	0.08
Raped	20	3.3	17	3.1	3	6.1	0.22

* *p<*0.05

** *p<*0.01

*** *p<*0.001

a *p*-value derived using Pearson's chi-square test; Fisher's exact test was used for cell counts less than 5; MSM, men who have sex with men.

**Table 11:** Prevalence of sexual behaviour stigma among transgender women as compared with MSM participants in Burkina Faso (*N=*541)

	Total		MSM		Transgender women		
				
	*n*	%	*n*	%	*n*	%	*χ*^2^*p*^a^
**Stigma from family/friends**							
Excluded by family	53	9.8	36	8.1	17	17.4	0.006**
Gossiped about by family	161	29.8	125	28.2	36	37.1	0.08
Rejected by friends	166	30.7	127	28.7	39	39.8	0.03*
**Perceived healthcare stigma**							
Afraid to seek healthcare	154	28.5	133	30.0	21	21.4	0.09
Avoided seeking healthcare	136	25.1	114	25.7	22	22.5	0.50
**Experienced healthcare stigma**							
Treated poorly at a healthcare centre	18	3.3	13	2.9	5	5.1	0.28
Gossiped about by healthcare worker	39	7.2	32	7.2	7	7.1	0.98
**Social stigma**							
Verbally harassed	209	38.7	152	34.4	57	58.2	<0.001***
Blackmailed	99	18.3	86	19.5	13	13.3	0.15
Physically hurt	67	12.5	40	9.1	27	27.8	<0.001***
Raped	52	9.7	38	8.6	14	14.3	0.09

* *p<*0.05

** *p<*0.01

*** *p<*0.001

a *p*-value derived using Pearson's chi-square test; MSM, men who have sex with men.

**Table 12:** Prevalence of sexual behaviour stigma among transgender women as compared with MSM participants in Côte d'Ivoire (*N=*1100)

	Total		MSM		Transgender women		
				
	*n*	%	*n*	%	*n*	%	*χ*^2^*p*^a^
**Stigma from family/friends**							
Excluded by family	119	10.8	74	9.3	45	14.7	0.01*
Gossiped about by family	386	35.1	240	30.2	146	47.7	<0.001***
Rejected by friends	265	24.1	165	20.8	100	32.7	<0.001***
**Perceived healthcare stigma**							
Afraid to seek healthcare	266	24.2	184	23.2	82	26.8	0.21
Avoided seeking healthcare	185	16.8	122	15.4	63	20.6	0.04*
**Experienced healthcare stigma**							
Treated poorly at a healthcare centre	35	3.2	22	2.8	13	4.3	0.21
Gossiped about by healthcare worker	104	9.5	72	9.1	32	10.5	0.48
**Social stigma**							
Verbally harassed	421	38.3	241	30.4	180	58.8	<0.001***
Blackmailed	215	19.6	141	17.8	74	24.2	0.02*
Physically hurt	165	15.1	82	10.4	83	27.3	<0.001***
Raped	158	14.4	76	9.6	82	26.8	<0.001***

* *p<*0.05

** *p<*0.01

*** *p<*0.001

a *p*-value derived using Pearson's chi-square test; MSM, men who have sex with men.

### Independent associations with HIV infection among transgender women

**Figure 1:**
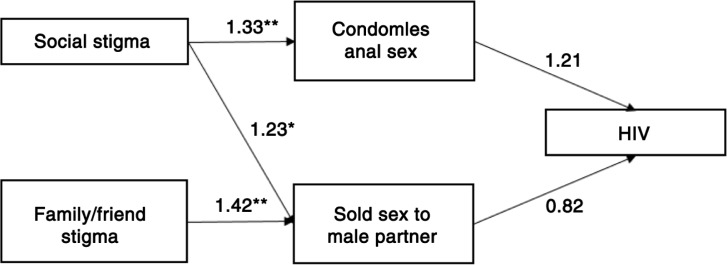
GSEM indicating adjusted odds ratios for associations between sexual behaviour stigma and HIV risk-related characteristics among transgender women in three West African nations (*N=*417). *p<0.05; **p<0.01.

The GSEM was used to analyze mediated associations of sexual behaviour stigma and HIV infection among transgender women. Country-specific models could not be used due to the limited number of transgender women and HIV cases by country. Those who were aware that they were currently living with HIV were removed (*n=*36) in order to eliminate potential bi-directionality of the association between knowledge of living with HIV and sexual behaviour stigma, resulting in a final sample of 417 transgender women. Each sexual behaviour stigma domain variable was conceptualized as an explanatory cause of HIV infection, and potential mediators included sexual positioning, number of recent receptive anal sex partners, condomless anal sex and engagement in sex work. Variables that were not statistically significant (*p<*0.05) were dropped from the model; however, HIV laboratory diagnosis was kept in order to describe associations of interest, and age and education were kept to control for potential confounding. In the final model, social stigma was positively associated with condomless anal sex at the last anal sex episode (*p<*0.01). In addition, social stigma and family/friend stigma were associated with selling sex to a male partner within the past 12 months (*p<*0.01) (Table 13; Figure 1). However, condomless anal sex and engagement in sex work were not found to be significantly associated with HIV infection.

**Table 13:** GSEM-adjusted associations with testing positive for HIV and potential mediators among transgender women in three West African nations (*N=*417)

Outcome variable	Explanatory variable	Adjusted odds ratio	95% confidence interval	*p*
Condomless anal sex, last anal sex episode	Social stigma	1.33	1.09, 1.62	0.004**
Sold sex to male partner, past 12 months	Social stigma	1.23	1.02, 1.50	0.03*
	Family/friend stigma	1.42	1.13, 1.79	0.003**
Positive for HIV	Condomless anal sex, last anal sex episode	1.21	0.63, 2.33	0.56
	Sold sex to male partner, past 12 months	0.82	0.44, 1.53	0.54

* *p*<0.05

** *p*<0.01; GSEM, generalized structural equation model; MSM, men who have sex with men.

*Note*: Model adjusts for age and education. Clustering is taken into account at the site level.

## Discussion

In these analyses of transgender women in three West African nations, we found a high burden of HIV, nearly three times that of MSM, who are known as a key population at risk for HIV infection. Previous studies among transgender women in the United States have pointed to higher levels of condomless anal sex and engagement in sex work as key risk factors for HIV transmission, driven potentially by underlying experiences or perceptions of stigma and economic marginalization [3,15,17]. In this sample, we found that sexual behaviour stigma from family/friends and broader social groups was positively and significantly associated with recent condomless anal sex as well as engagement in sex work, which could help to partially explain the disproportionate burden of HIV among this population.

Another important finding is the relatively large proportion of participants who identified as a woman or transgender as part of these RDS-generated samples of individuals born male who have sex with men. Our findings highlight that transgender women comprise a distinct population from that of MSM, with increased levels of stigma and risk of HIV infection. Of further note is the relationship between sexual positioning and gender identity, which suggests that transgender women are more likely to engage exclusively in receptive anal sex. One possibility is that some participants may have confused questions about gender identity with questions about sexual positioning (e.g., those who bottom being more likely to indicate female gender). However, we used a two-step process for gender assessment, which is the method recommended by the UCSF Center of Excellence for Transgender Health [39]. In addition, our analyses further indicate that, overall, transgender women are equally likely as MSM to engage in sexual position versatility (i.e., to practice both receptive and insertive anal sex). This sexual position versatility has implications for increasing network-level HIV transmission risks, in that it enables partners who are newly infected through receptive anal sex to transmit HIV efficiently to new partners when they are the insertive partner [4,40]. Taken together, these results suggest that participants in this study defined their gender identity separately from their sexual practices and that sexual practices alone cannot explain the disproportionately high burden of HIV among transgender women.

In a sensitivity analysis, we included the 210 individuals in Togo and Burkina Faso who reported their gender as intersex (*n=*208) or other (*n=*2) as a separate category in the bivariate analyses. We found that these individuals had a relatively similar distribution of sociodemographic characteristics and experience of stigma as that of transgender women; however, they were much more likely to report versatile sexual positioning (73% in Togo and 76% in Burkina Faso). The HIV prevalence of these individuals was 21% in Togo and 4% in Burkina Faso, which was similar to the prevalences of 19% and 5% observed among transgender women in each country, respectively. Based on these findings, it appears that those who identify as intersex may have similar HIV risks to transgender women, reinforcing the need to better understand gender diversity across these settings.

Stigma likely plays an important role in facilitating risk for HIV transmission in this population. After adjusting for sociodemographic factors and clustering within study sites, we found that social and family/friend stigma was associated with engagement in sex work, and social stigma was associated with increased report of condomless anal sex at the last anal sex episode. Although condomless anal sex and engagement in sex work were not found to be empirically associated with prevalent HIV infection in this sample, they nonetheless serve as important risk factors for HIV transmission [41,42]. Sample size limitations and underreporting of sexual risk practices due to social desirability bias in face-to-face interviews, as well as the timing of HIV infection as measured in relation to risk practices (i.e., condom use at last anal sex episode), could explain the lack of finding of an association here.

There are additional limitations of this study to consider. First, temporality could not be established with the use of cross-sectional data. However, we removed those who self-reported living with HIV from our final multivariable model, reducing the likelihood that knowledge of HIV status affected sexual behaviour stigma or sexual risk practices. In addition, transgender women face multiple levels of intersecting stigma, including gender identity stigma and HIV stigma. We measured only sexual behaviour stigma; however, these measures had good validity in this sample and were found to be significant in predicting risk factors for HIV infection among those unaware of living with HIV. This intercategorical approach to assessing intersectionality of sexual behaviour stigma, by focusing analyses on categories of gender identity, is an approach that has been recommended in previous work [26]. Another limitation is that a large proportion of participants in Togo had missing data for sexual positioning (55%), and these results should be interpreted with caution. Because transgender women were recruited via predominantly MSM social networks, we may have failed to reach transgender women not linked to the MSM community via social or sexual networks. Due to sample size limitations, it was not possible to run separate GSEMs for each country. Because Côte d'Ivoire accounted for roughly half of the study sample and the majority of prevalent HIV cases, we note that the findings from the GSEM were driven primarily by data from Côte d'Ivoire. Finally, there may have been differences in culture, time period and access to healthcare across settings that were not explored. For example, Abidjan, Bobo-Dioulasso and Lomé have areas that are more culturally open to and accepting of MSM than other regions within the same country and, although we adjusted for study site in the GSEM, we did not assess the impact of these site-specific factors on stigma or risk for HIV.

## Conclusion

The World Health Organization and UNAIDS recently called for a reduction of stigma towards MSM and transgender women to reduce HIV transmission in these key populations [43,44]. Overall, these data suggest that sexual behaviour stigma perpetuated against transgender women is widespread in Togo, Burkina Faso and Côte d'Ivoire and is even more extreme than stigma directed towards MSM. This stigma also has a significant impact on HIV risk-related behaviours and will need to be addressed as part of a comprehensive HIV prevention strategy. Empirical data among transgender women across sub-Saharan Africa are historically limited. However, these findings indicate that transgender women are present in substantial numbers in West Africa. They are a distinct population from MSM, and this distinction is not defined exclusively by sexual positioning. Continued efforts are needed to improve our understanding of gender identity and to better meet the HIV treatment, care and prevention needs of transgender women across the continent.
